# Supplementary material for: Aberrant upregulation of the glycolytic enzyme PFKFB3 in CLN7 neuronal ceroid lipofuscinosis
Source: Nat Commun. 2022 Jan 27;13:536. doi: 10.1038/s41467-022-28191-1 (PMC8795187; doi:10.1038/s41467-022-28191-1)
Supplement: Supplementary file 1 — Supplementary Information [file 41467_2022_28191_MOESM1_ESM.pdf]

Supplementary Information

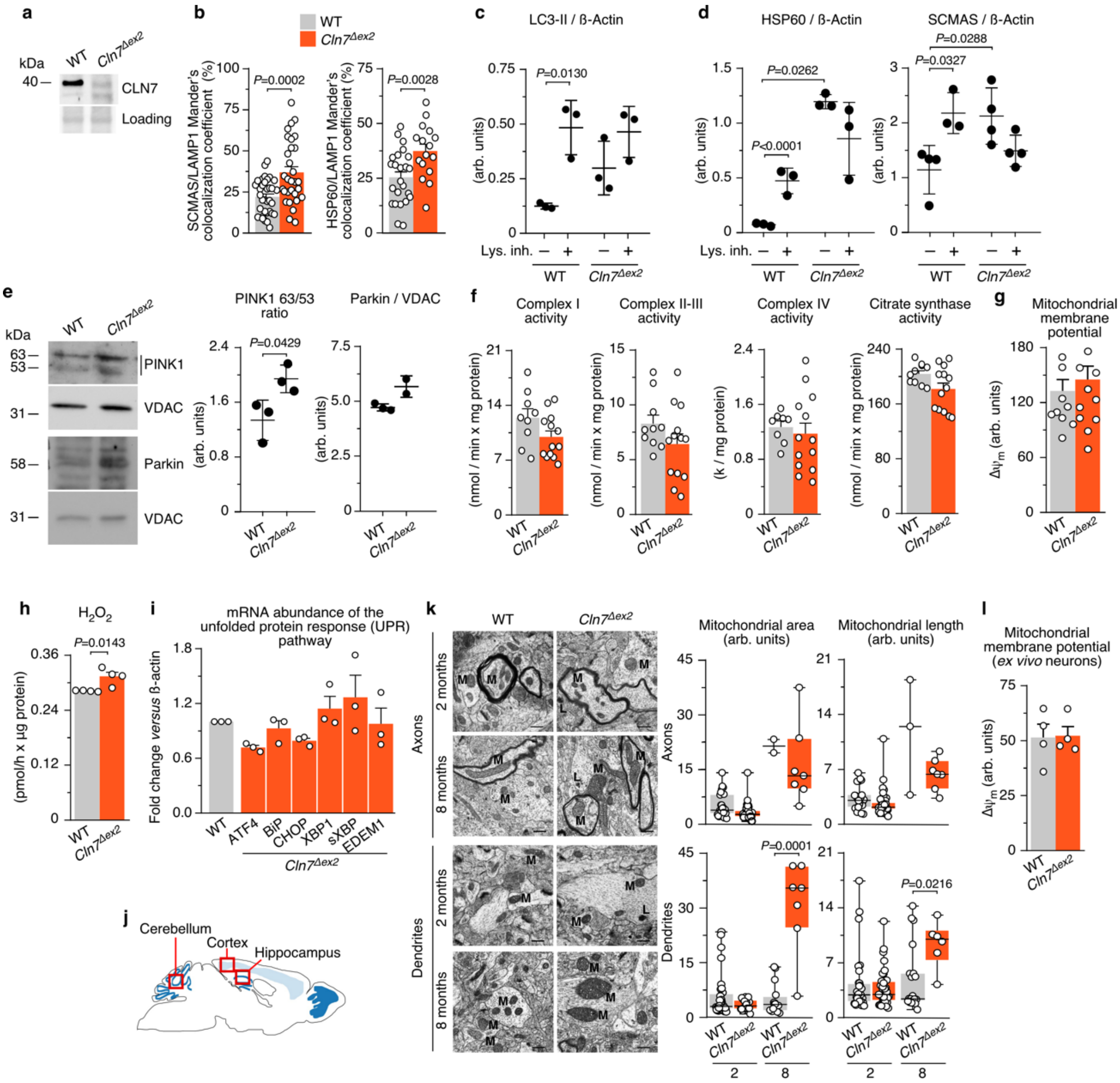

Supplementary Fig. 1. Extended data to figure 1 on failure in autophagy causes accumulation of structural and functionally impaired mitochondria in *Cln7*$^{\Delta ex2}$ mouse.

(**a**) Cln7 representative Western blot analysis in primary neurons (loading control Ponceau staining).

(b) Co-localization analyses of SCMAS/LAMP1 and HSP60/SCMAS in primary neurons of images shown in Fig. 1a (Mander's coefficient) . Data are mean ± S.E.M. of n≥13 neurons from n=3 independent samples.

(c) Densitometric quantification of the Western blot bands shown in Fig. 1b and the replicas. Data are mean ± S.D. from n=3 independent samples.

(d) Densitometric quantification of the Western blot bands shown in Fig. 1c and the replicas. Data are mean ± S.D. from n=3 (HSP60), n=4 (SCMAS) independent samples.

(e) PINK1 Western blot analysis of 63 and 53 kDa bands, and Parkin Western blot in primary neuronal mitochondria (VDAC, loading control) (left). Densitometric quantification of the Western blot bands and the replicas (right). Data are mean ± S.D. from n=3 or n=2 (Parkin-*Cln7*$^{\Delta ex2}$) independent samples.

(f) Activities of the mitochondrial respiratory chain complexes I, II-III and IV, and citrate synthase, in cell homogenates from primary neurons. Data are mean ± S.E.M. from n≥9 independent samples.

(g) Monitorization of mitochondrial membrane potential ($\Delta\psi_m$) in primary neurons. . Data are mean ± S.E.M. from n≥11 independent samples.

(**h**) H_2O_2 abundance as assessed by AmplexRed fluorescence in primary neurons (n=4). Data are mean ± S.E.M. from n=4 independent samples.

(i) ATF4, BiP, CHOP, total XBP1 and spliced XBP1 (sXBP1) and EDEM1 mRNA analysis by RT-qPCR in primary neurons. Data are mean ± S.E.M. from n=3 independent samples. (values normalized *versus* ß-actin).

(j) Brain schema indicating the location of analyzed areas in cerebellum, cortex and hippocampus.

(k) Representative electron microscopy images (left) and analyses of mouse brain cortex mitochondrial area and length (right). Data are in box plots (the box extends from the 25th to 75th percentiles, the horizontal line indicates the median, and the whiskers go down to the smallest value and up to the largest) from n≥2 mitochondria per condition . Scale bar, 500 nm. (M, mitochondria; L, lysosome).

(l) Monitorization of $\Delta\psi_m$ in freshly isolated neurons from mouse brain cortex. Data are mean ± S.E.M. from n=4 animals of 6 months-old).

Statistical analyses were performed by two tailed Student's t test (**b,e**,**f**,**g**,**h,i,k,l**) or one-way ANOVA followed by Tukey's post-hoc tests (**c,d**). Source data are provided as a Source Data file.

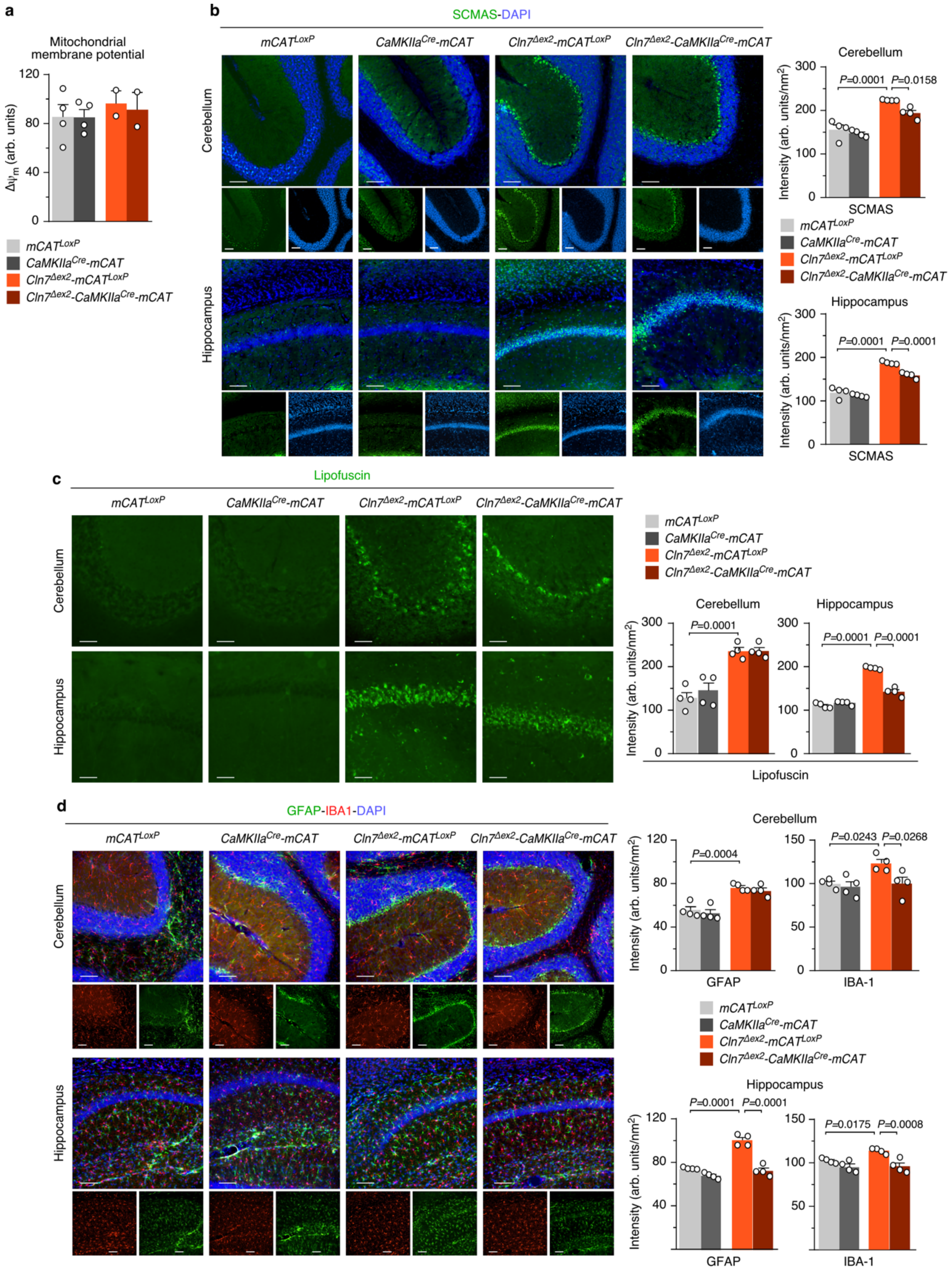

Supplementary Fig. 2. Extended data to figure 2 on increased generation of mitochondrial ROS by neurons accounts for impaired mitochondrial accumulation and hallmarks of CLN7 disease in *Cln7*$^{\Delta ex2}$ mouse in vivo.

(a) Monitorization of $\Delta\psi_m$ in primary neurons. Data are the mean ± S.E.M. from n=4 ($mCAT^{LoxP}$; $CaMKIIa^{Cre}$-mCAT), n=2 ($Cln7^{\Delta ex2}$-$mCAT^{LoxP}$, $Cln7^{\Delta ex2}$-$CaMKIIa^{Cre}$-mCAT) independent samples.

(b,c,d) Representative images of SCMAS, lipofuscin, GFAP and IBA-1 immunohistochemical analysis of the mouse brain hippocampus and cerebellum. Data are mean ± S.E.M. from 3 serial slices per 3 months-old mouse (n=4 mice; one-way ANOVA followed by post-hoc Tukey). Scale bar, 100 µm.

Source data are provided as a Source Data file.

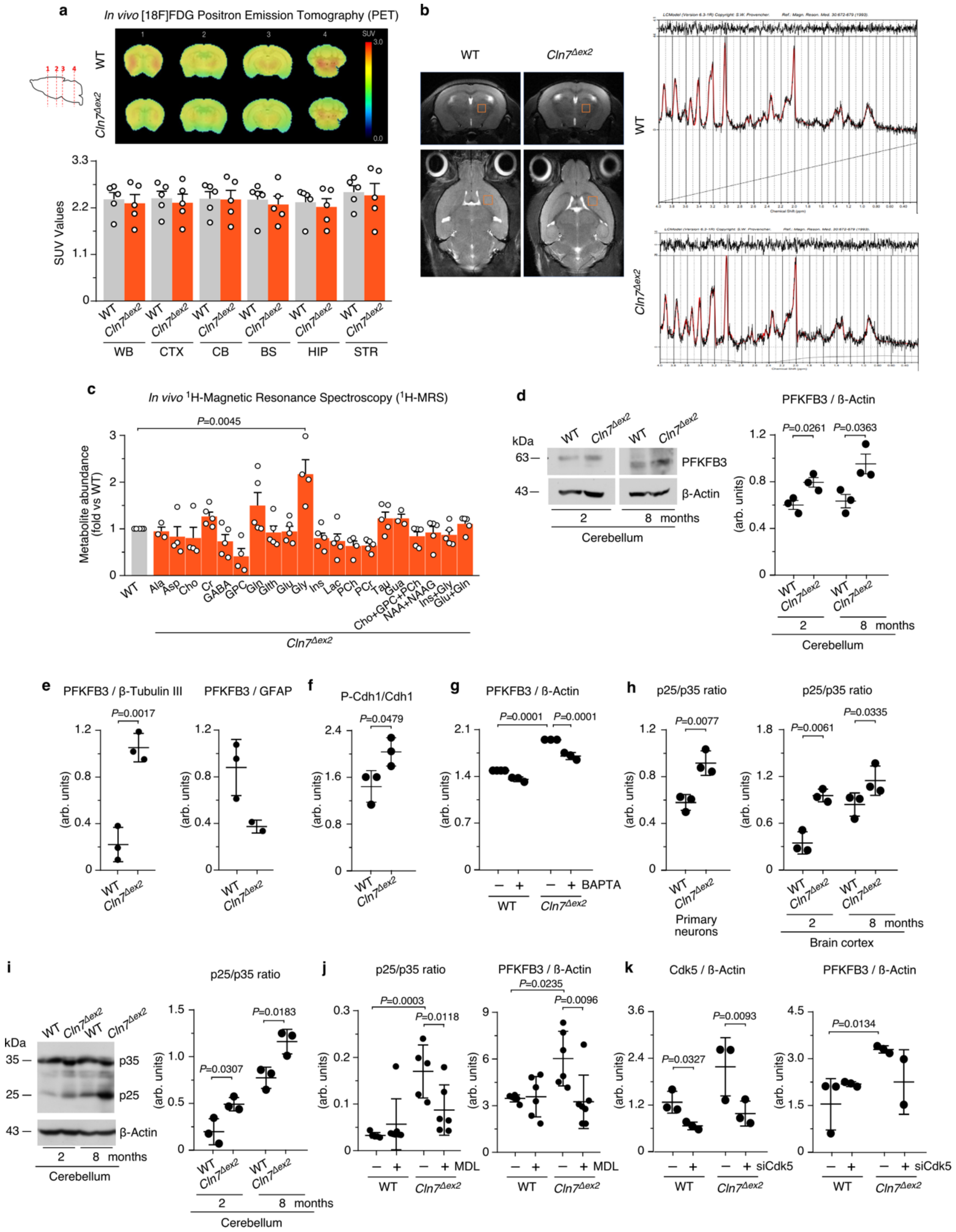

Supplementary Fig. 3. Extended data to figure 3 on upregulation of PFKFB3 protein and activity *via* a Ca^{2+}/calpain/Cdk5 pathway sustains a high glycolytic flux in *Cln7*$^{\Delta ex2}$ neurons.

(a) *In vivo* 2-[^{18}F]fluoro-2-deoxy-D-glucose ([^{18}F]FDG) uptake, as analyzed by positron emission tomography (PET) in whole brain (WB), cortex (CTX), cerebellum (CB),

brain stem (BS), hippocampus (HIP) and striatum (STR) in wild type and *Cln7*$^{\Delta ex2}$ mice. Data are mean ± S.E.M. from n=5 animals of 5 months-old.

(b) Left-hand panel, MR brain images (transverse on top, coronal below) showing a typical location of an acquired voxel (colored box at the right striatum); right-hand panel, typical ^{1}H-MRS data showing acquired (black lines) and fitted (red lines) spectra.

(c) Data of the in vivo brain ^{1}H-MRS analysis. Data are mean ± S.E.M. from n=5 animals of 3-months-old(analyzed area, striatum). (GPC, glycerophosphocholine; Ins, myo-inositol; PCh, phosphocholine; Gua, guanidoacetate).

(d) Representative Western blot showing PFKFB3 protein in cerebellum (ß-actin, loading control) (left); densitometric quantification of these bands and replicas (right). Data are mean ± S.D. from n=3.

(e) Densitometric quantification of the western blot bands and replicas shown in Fig. 3e. Data are mean ± S.D. from n=3 animals of 6 months-old.

(f,g,h) Densitometric quantification of the western blot bands shown in Figs. 3h (**e**), 3j (**f**) and 3l (**g**), and replicas. Data are mean ± S.D. from n=3 independent samples .

(i) Representative western blot showing p35 and its cleavage product p25 in cerebellum (ß-actin, loading control) (left); densitometric quantification of these bands and replicas (right) Data are mean ± S.D. from n=3 animals.

(j,k) Densitometric quantification of the western blot bands shown in Fig. 3m (**j**), Fig. 3n (**k**) and replicas. Data are mean ± S.D. from n≥4 (**i**), n=3 (**j**) independent samples. Statistical analyses were performed by one-way ANOVA followed by Tukey's (**g,j**) or DMS's (**j**) post-hoc tests, or two tailed Student's t test (**a,c,d,e,f,h,i**). Source data are provided as a Source Data file.

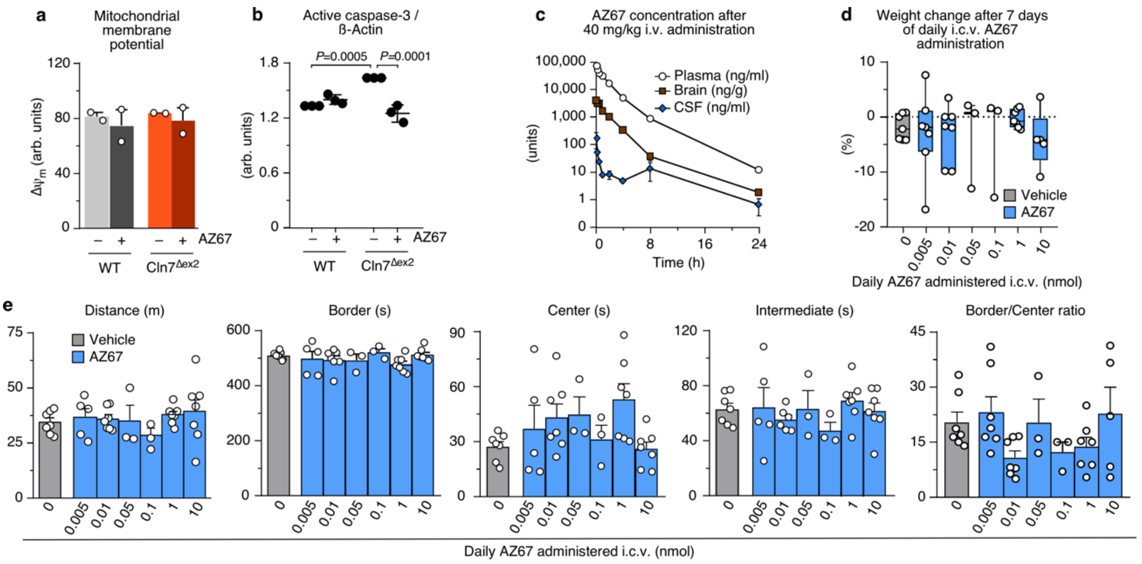

Supplementary Fig. 4. Extended data to figure 4 on pharmacological targeting PFKFB3 restores mitochondrial alterations of Cln7$^{\Delta ex2}$ disease in vivo.

(**a**) Monitorization of $\Delta\psi_m$ in primary neurons. Data are mean ± S.E.M. from n=2 independent samples.

(b) Densitometric quantification of the western blot bands shown in Fig. 4d replicas. Data are mean ± S.D. fromn=3 independent samples.

(c) Time course evaluation of AZ67 concentration in plasma, brain and cerebrospinal fluid after the intravenous administration of 40 mg/kg of AZ67 in C57Bl/6 mice. Data are mean ± S.E.M. from n=3 animals. Related to Figs. 4e-g.

(d) Dose response evaluation of the weight fluctuations after 7 days of a daily intracerebroventricular injection of AZ67 (1 nmol/mouse).

Data are in box plots (the box extends from the 25th to 75th percentiles, the horizontal line indicates the median, and the whiskers go down to the smallest value and up to the largest) from n≥3 animals of 2 months-old. Related to Figs. 4e-g.

(e) Dose response evaluation of the open field test after 7 days of a daily intracerebroventricular injection of AZ67 (1 nmol/mouse). Total distance, time spend in border, center and intermediate zone, and the ratio of border to center time are

represented. Data are mean ± S.E.M. from n≥3 animals of 5 months-old animals.

Related to Figs. 4e-g.

Statistical analyses were performed by one-way ANOVA followed by Tukey's post-hoc tests. Source data are provided as a Source Data file.

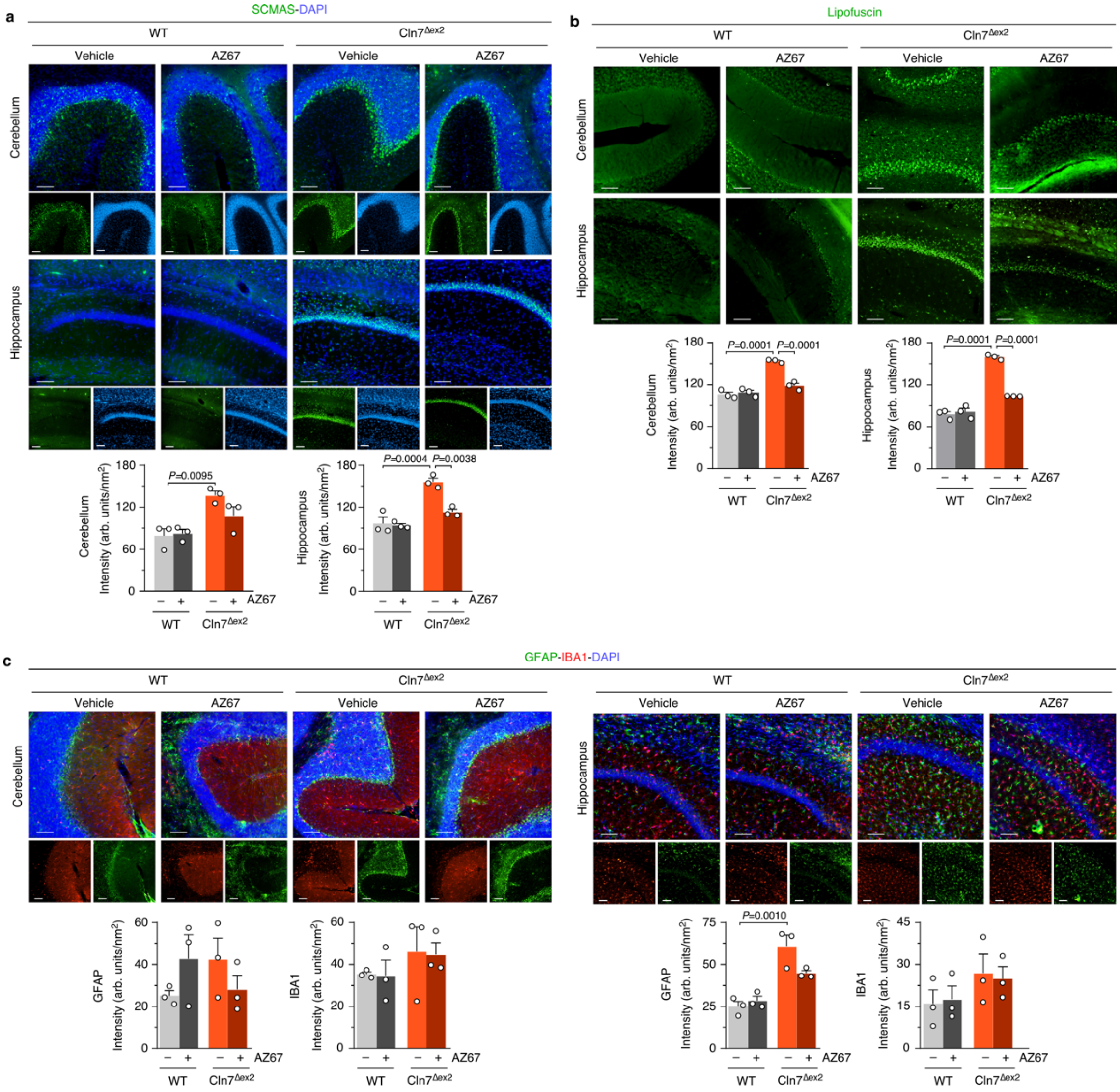

Supplementary Fig. 5. Extended data to figure 5 on pharmacological targeting PFKFB3 restores hallmarks of Cln7$^{\Delta ex2}$ disease in vivo.

(a,b,c) Immunohistochemical analysis of the cerebellum and hippocampus of 3 months-old mice after 2 months of a daily intracerebroventricular administration of AZ67 (1 nmol/mouse) against SCMAS (**a**), lipofuscin (autofluorescence) (**b**), GFAP and IBA-1 (**c**). Data are mean ± S.E.M. from the quantification of 3 serial slices per mouse in n=3 mice (one-way ANOVA followed by Tukey's post-hoc test). Scale bar, 100 µm. Source data are provided as a Source Data file.

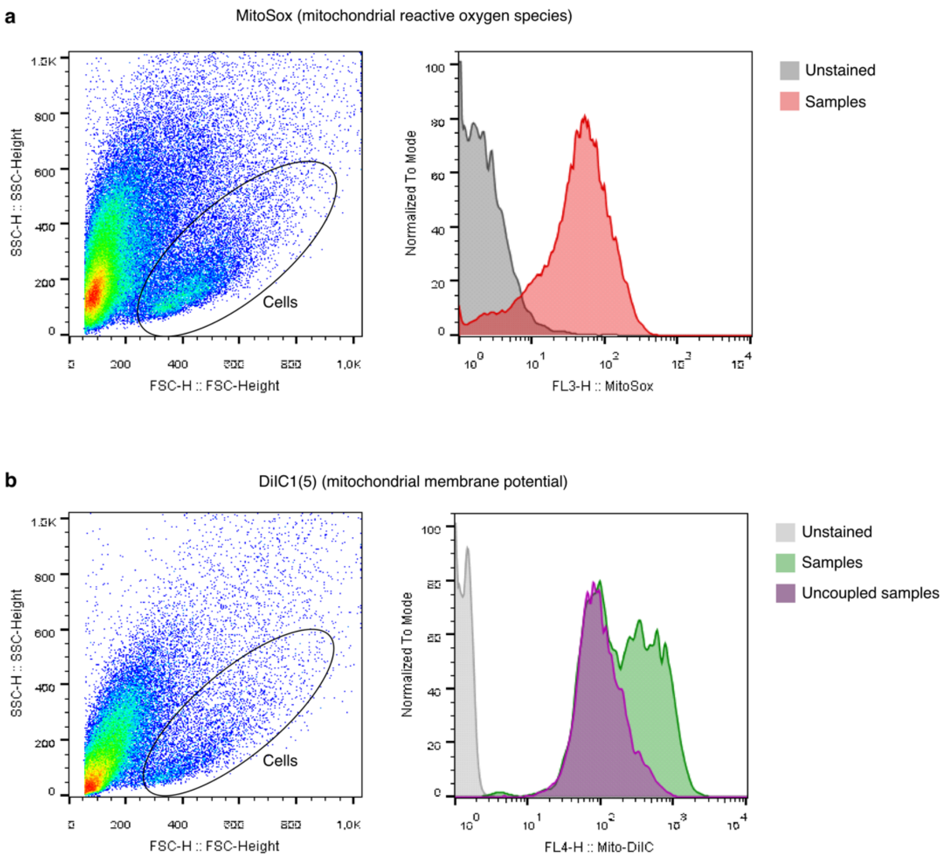

Supplementary Fig. 6. Flow cytometry workflows.

(a) MitoSox, to assess mitochondrial reactive oxygen species (mROS) in brain cells. The median of the selected population was used.

(b) DiIC1(5), to assess mitochondrial membrane potential ($\Delta\psi_m$) in brain cells. $\Delta\psi_m$ is calculated subtracting, to the median of the selected population, the median of uncoupled population.

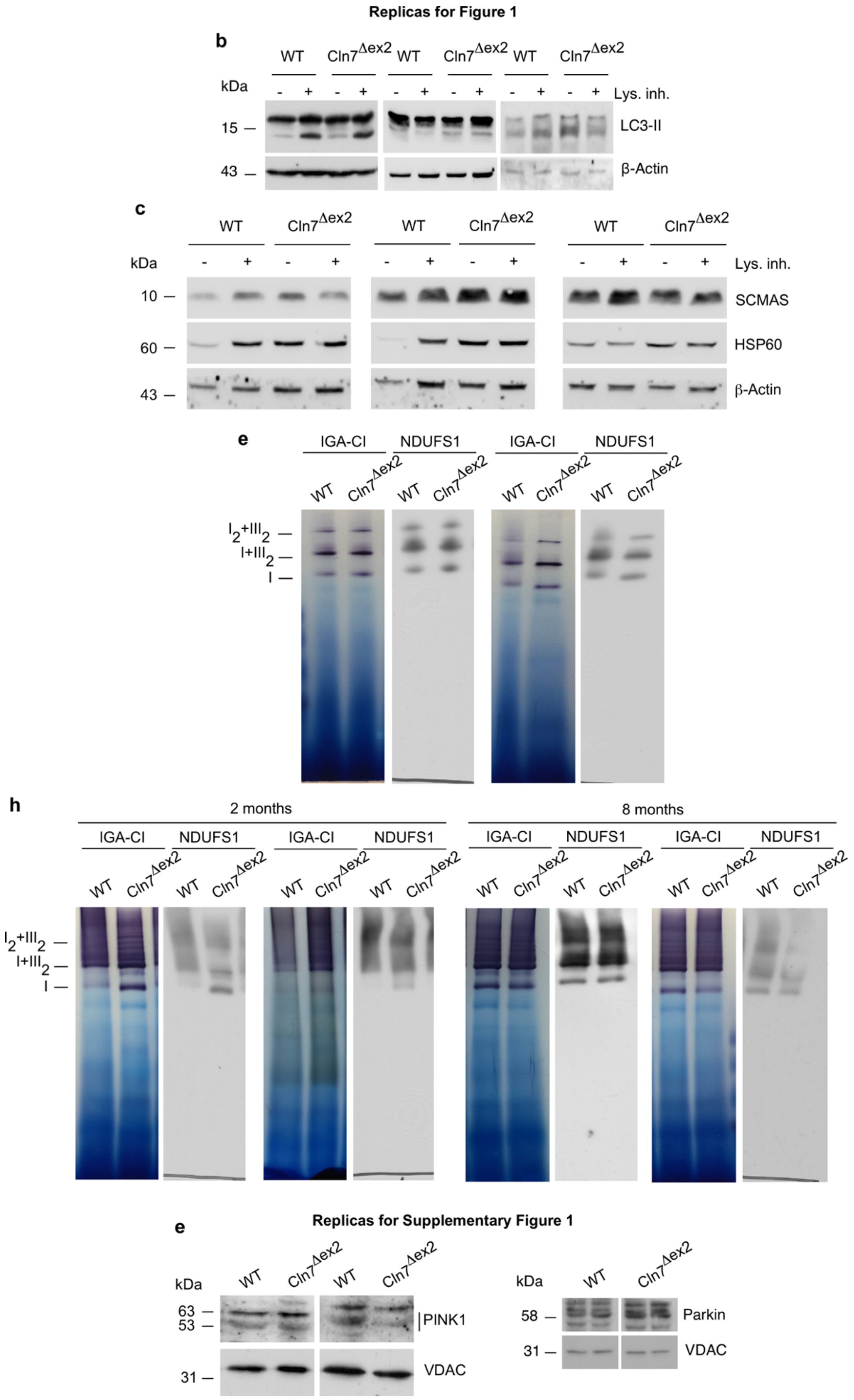

Supplementary Figure 7. Western blots replica for Fig. 1 and Supplementary Fig. 1.

Replicas for Figure 3

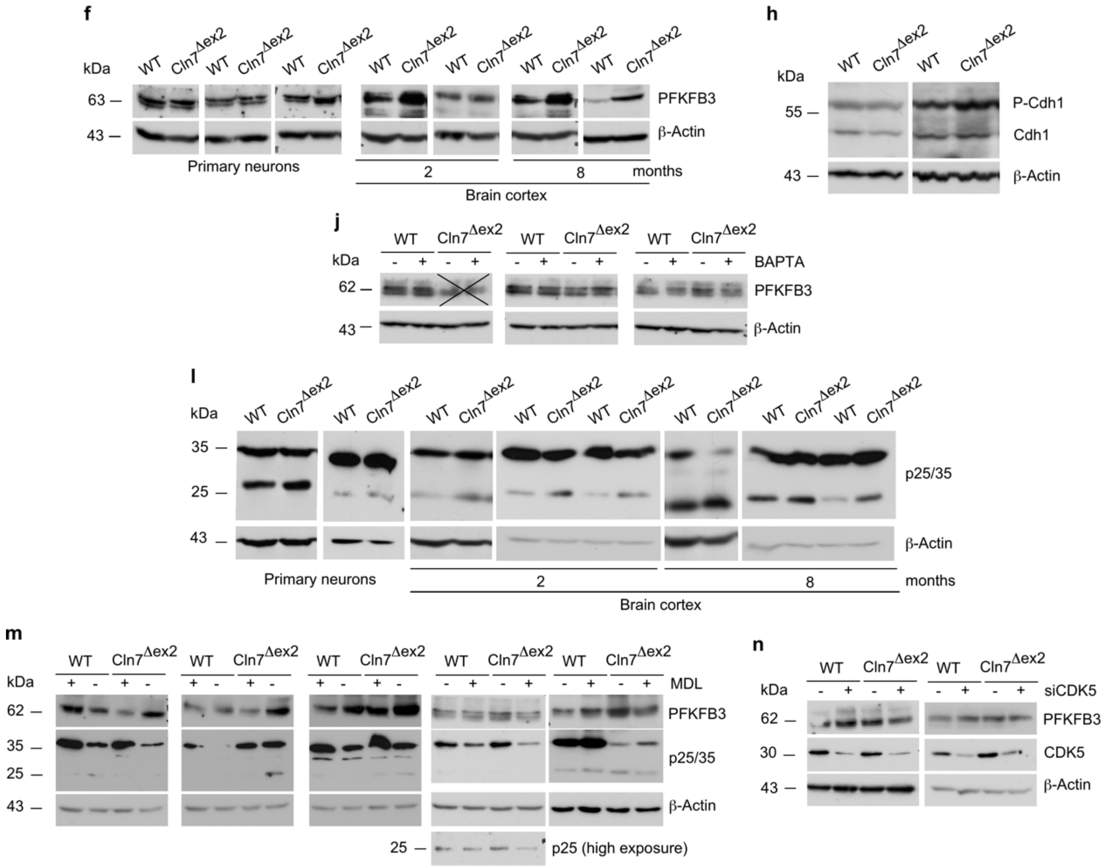

Replicas for Supplementary Figure 3

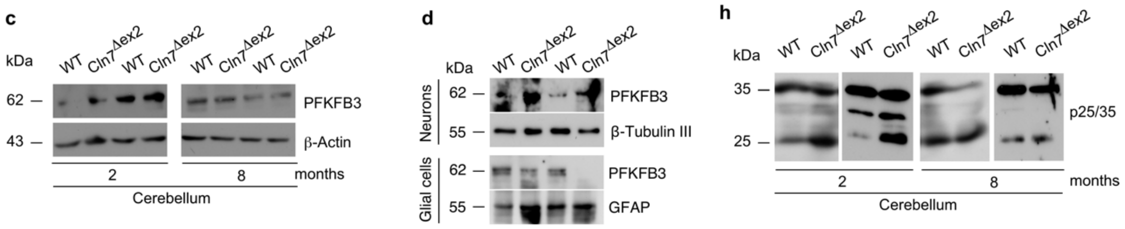

Replicas for Figure 4

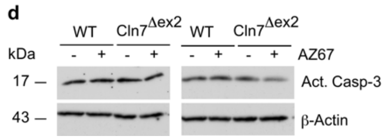

Supplementary Figure 8. Western blots replica for Fig. 3, Supplementary Fig. 3 and Fig. 4.

Reagent or Resource	Source	Identifier
Antibodies		
ATP-C	Abcam	Cat# ab181243
VDAC	Millipore	Cat# PC548, RRID:AB_2257155
HSP60	Abcam	Cat# ab46798, RRID:AB_881444
PINK1	Santa Cruz Biotechnology	Cat# sc-33796, RRID:AB_2164259
NDUFS1 (E-20)	Santa Cruz Biotechnology	Cat# sc-50132, RRID:AB_2298398
PFKFB3 (Clone 3F3)	Novus Biologicals	Cat# H00005209-M08, RRID:AB_1146566
p35/25 (Clone C64B10)	Cell Signaling Technology	Cat# 2680, RRID:AB_1078214
Cleaved Caspase-3 (Asp175)	Cell Signaling Technology	Cat# 9661, RRID:AB_2341188
β-Actin	Sigma	Cat# A5441, RRID:AB_476744
CDK5 (J-3)	Santa Cruz Biotechnology	Cat# sc-6247, RRID:AB_627241
LAMP1	DSHB	Cat# 1d4b, RRID:AB_2134500
Parkin	Santa Cruz Biotechnology	Cat# sc-32282, RRID:AB_628104
OCT4	Abcam	Cat# ab19857, RRID:AB_445175
SOX2	R&D Systems	Cat# AF2018, RRID:AB_355110
Nanog	Abcam	Cat# ab21624, RRID:AB_446437
Tra-1-60	R&D Systems	Cat# MAB1295, RRID:AB_2185575
TUJ1	R&D Systems	Cat# MAB1195, RRID:AB_357520
b-Tubulin III	Abcam	Cat# ab18207, RRID:AB_444319
ATP5A (Clone 15H4)	Abcam	Cat# ab14748, RRID:AB_301447
GFAP (Clone G-A-5)	Sigma	Cat# G6171, RRID:AB_1840893
Iba1	Wako	Cat# 019-19741, RRID:AB_839504
Nestin (Clone 10C2)	Thermo Fisher	Cat# MA1-110, RRID:AB_2536821
LC3B	Cell Signaling Technology	Cat# 2775, RRID:AB_915950
Rabbit-HRP	Santa Cruz Biotechnology	Cat# sc-2030, RRID:AB_631747
Rabbit-HRP	Biorad	Cat# 170-6515, RRID:AB_11125142
Mouse-HRP	Biorad	Cat# 170-6516, RRID:AB_11125547
Goat-HRP	Abcam	Cat# ab6741, RRID:AB_955424
Cy2 goat anti-mouse	Jackson Immuno-Research	Cat# 115-225-003, RRID:AB_2338740
Cy3 goat anti-rabbit	Jackson Immuno-Research	Cat# 111-165-003, RRID:AB_2338000
Cy3 streptavidin	Jackson Immuno-Research	Cat# 016-160-084, RRID:AB_2337244
CD45	BD	Cat# 553076, RRID:AB_394606
b-Tubulin III	Sigma	Cat# T2200, RRID:AB_262133
GFAP	Millipore	Cat# AB5541, RRID:AB_177521
Alexa 488 donkey anti-rabbit	Molecular Probes	Cat# A-31573, RRID:AB_2536183
Alexa 647 goat anti-rat	Thermo Fisher	Cat# A-21247, RRID:AB_141778
Alexa 568 goat anti-mouse	Invitrogen	Cat# A-11031, RRID:AB_144696
Biotin anti-mouse	Vector	Cat# BA-2000, RRID:AB_2313581
Alexa 488 goat anti-rabbit	Molecular Probes	Cat# A-11008, RRID:AB_143165
Chemicals, Peptides and Recombinat Proteins		
AZ PFKFB3 67	Tocris	Cat# 5742/10
MDL-28170	Sigma	Cat# M6690
BAPTA	Sigma	Cat# A1076
Critical Commercial Assays		
GenElute Mammalian Total RNA Miniprep Kit	Sigma	Cat# RTN70
Power SYBR Green RNA-to-CT 1-Step kit	Applied Biosystems	Cat# 4391178
Ad5CMVeGFP	University of Iowa Viral Vector Core	Cat# U of Iowa-4 Ad5CMVeGFP
Ad5CMVCre-eGFP	University of Iowa Viral Vector Core	Cat# U of Iowa-1174 Ad5CMVCre-eGFP
Neurobasal-A	Thermo Fisher	Cat# 10888-022
B27	Thermo Fisher	Cat# 17504001
Adult Brain Dissociation Kit	Miltenyi	Cat# 130-107-677, RRID: SCR_020295
Neuron Isolation Kit, mouse	Miltenyi	Cat# 130-115-39
siCDK5	Thermo Fisher	Cat# s201147
siControl	Thermo Fisher	Cat# 4390843
Lipofectamine RNAiMAX	Thermo Fisher	Cat# 13778075
NativePAGE Novex 3-12%	Thermo Fisher	Cat# BN1001BOX
MitoSOX	Thermo Fisher	Cat# M36008
AmplexRed	Thermo Fisher	Cat# A22177
DiIC1 (5) Assay Kit	Thermo Fisher	Cat# M34151
Fura-2 AM	Thermo Fisher	Cat# F1201
Pierce BCA Protein Assay Kit	Thermo Fisher	Cat# 23225
Software and Algorithms		
Seahorse Wave Desktop Software 2.6.1.56	Agilent	RRID:SCR_014526
ANY-Maze	HUGO Basile	RRID:SCR_014289
ImageJ	https://imagej.net/	RRID:SCR_003070
Tecnai Spirit TEM	FEI	
Harmony 4.0	Perkin Elmer	
Adobe Photshop CC	Adobe	RRID:SCR_014199
Prism v8	GraphPad	RRID:SCR_002798
IBM SPSS		RRID:SCR_019096
FlowJo v10		RRID:SCR_008520
Fluoview FV10-ASW	Olympus	RRID:SCR_014215

Table RRID Identifiers. Information on commercial sources and catalog numbers of antibodies, peptides, plasmids, kits, software and algorithms.
