# Supplementary material for: Aberrant upregulation of the glycolytic enzyme PFKFB3 in CLN7 neuronal ceroid lipofuscinosis
Source: Nat Commun. 2022 Jan 27;13:536. doi: 10.1038/s41467-022-28191-1 (PMC8795187; doi:10.1038/s41467-022-28191-1)
Supplement: Supplementary file 3 — Description of Additional Supplementary Files [file 41467_2022_28191_MOESM3_ESM.docx]

Description of Additional Supplementary Files

**Title: Supplementary movie 1.**

Description: Representative video of 5 months-old wild type mice after 2 months of a daily intracerebroventricular administration of vehicle.

**Title: Supplementary movie 2.**

Description: Representative video of 5 months-old wild type mice after 2 months of a daily intracerebroventricular administration of AZ67 (1 nmol/mouse).

**Title: Supplementary movie 3.**

Description: Representative video of 5 months-old *Cln7^∆ex2^* mice after 2 months of a daily intracerebroventricular administration of vehicle.

**Title: Supplementary movie 4.**

Description: Representative video of 5 months-old *Cln7^∆ex2^* mice after 2 months of a daily intracerebroventricular administration of AZ67 (1 nmol/mouse).
