# Supplementary material for: Aberrant upregulation of the glycolytic enzyme PFKFB3 in CLN7 neuronal ceroid lipofuscinosis
Source: Nat Commun. 2022 Jan 27;13:536. doi: 10.1038/s41467-022-28191-1 (PMC8795187; doi:10.1038/s41467-022-28191-1)
Supplement: Supplementary file 8 — Reporting Summary [file 41467_2022_28191_MOESM8_ESM.pdf]

Corresponding author(s): Irene López-Fabuel
Juan Pedro Bolaños

Last updated by author(s): Oct 25, 2021

Reporting Summary

Nature Research wishes to improve the reproducibility of the work that we publish. This form provides structure for consistency and transparency in reporting. For further information on Nature Research policies, see our Editorial Policies and the Editorial Policy Checklist.

Statistics

For all statistical analyses, confirm that the following items are present in the figure legend, table legend, main text, or Methods section.

n/a	Confirmed	
[ ]	[x]	The exact sample size (*n*) for each experimental group/condition, given as a discrete number and unit of measurement
[ ]	[x]	A statement on whether measurements were taken from distinct samples or whether the same sample was measured repeatedly
[ ]	[x]	The statistical test(s) used AND whether they are one- or two-sided<br>*Only common tests should be described solely by name; describe more complex techniques in the Methods section.*
[ ]	[x]	A description of all covariates tested
[x]	[ ]	A description of any assumptions or corrections, such as tests of normality and adjustment for multiple comparisons
[ ]	[x]	A full description of the statistical parameters including central tendency (e.g. means) or other basic estimates (e.g. regression coefficient) AND variation (e.g. standard deviation) or associated estimates of uncertainty (e.g. confidence intervals)
[ ]	[x]	For null hypothesis testing, the test statistic (e.g. *F*, *t*, *r*) with confidence intervals, effect sizes, degrees of freedom and *P* value noted<br>*Give P values as exact values whenever suitable.*
[x]	[ ]	For Bayesian analysis, information on the choice of priors and Markov chain Monte Carlo settings
[x]	[ ]	For hierarchical and complex designs, identification of the appropriate level for tests and full reporting of outcomes
[x]	[ ]	Estimates of effect sizes (e.g. Cohen's *d*, Pearson's *r*), indicating how they were calculated

Our web collection on statistics for biologists contains articles on many of the points above.

Software and code

Policy information about availability of computer code

Data collection	CellQuest Pro v5, Tecnai™ Spirit TEM, QuantaSmart for TriCarb 5.1 (Perkin Elmer), AMi-maze® interface and ANY-maze® 5.33 software, Seahorse Wave Desktop Software 2.6.1.56, BioRad CFX Maestro 1.1, Harmony 4.9, Fluoview FV10-ASW
Data analysis	Adobe Photoshop CC, ImageJ 1.48V, Paint-A-Gate ™ PRO, GraphPad Prism v8.0, IBM SPSS 23.0, FlowJo v10, Microsoft Excel (Microsoft 365) , Fluoview FV10-ASW

For manuscripts utilizing custom algorithms or software that are central to the research but not yet described in published literature, software must be made available to editors and reviewers. We strongly encourage code deposition in a community repository (e.g. GitHub). See the Nature Research guidelines for submitting code & software for further information.

Data

Policy information about availability of data

All manuscripts must include a data availability statement. This statement should provide the following information, where applicable:

- Accession codes, unique identifiers, or web links for publicly available datasets
- A list of figures that have associated raw data
- A description of any restrictions on data availability

Source data for each figure are provided with this paper: the raw data and original immunoblots that support all the figures and findings of this study are available in the Source Data file. The datasets generated and analysed during the current study are available from the corresponding author on reasonable request.

Field-specific reporting

Please select the one below that is the best fit for your research. If you are not sure, read the appropriate sections before making your selection.

- [x] Life sciences
- [ ] Behavioural & social sciences
- [ ] Ecological, evolutionary & environmental sciences

For a reference copy of the document with all sections, see nature.com/documents/nr-reporting-summary-flat.pdf

Life sciences study design

All studies must disclose on these points even when the disclosure is negative.

Sample size	No statistical methods were used to determine sample size prior to experiments. Sample sizes were decided by the previous experience of the group in in vitro molecular experiments and in vivo experiments and/or on previously published similiar experiments.<br>Exact information on the sample numbers being analyzed can be found in Figure legends and in Supplementary Information.<br>The majority of biochemical assays were repeated at least three times in order to derive statistical information such as error bars, p values and significance.
Data exclusions	No data were excluded from analyses.
Replication	In vitro: experiments were done from 3 to 5 times with independent biological samples and the necessary technical replicates for each technique (tipically, 4-6 replicas), in order to reproduce the results found. In vivo: the sample size of the behavioral studies was higher than in the in vitro experiments given the variability in the parameters measured in order to confirm a reliable result. This information has been added in the Statistical section of the manuscript. All attempts at replication were successfull.
Randomization	For all mouse experiments, animals were chosen based on genotypes. Aged-matched wild-type and mutant littermates were compared to minimize variance in age, genetic background and environment. Then ageneral method of randomization to assign experimental groups was not performed because all experiments were conducted with appropiate positive and negative controls, therefore it was not applicable.<br>For in vitro studies, randomization is not applicable as cells with different treatments or genetic knockdown cannot be randomized. However, for imaging experiments, cells were chosen at random within each condition.
Blinding	Blinding was not considered to be necessary in biochemical, blotting and imaging experiments because they were analyzed in exactly the same manner.<br>For in vivo analysis, all experiments were done by experienced researchers blind for the experimental conditions.

Reporting for specific materials, systems and methods

We require information from authors about some types of materials, experimental systems and methods used in many studies. Here, indicate whether each material, system or method listed is relevant to your study. If you are not sure if a list item applies to your research, read the appropriate section before selecting a response.

Materials & experimental systems

n/a	Involved in the study	
[ ]	[x]	Antibodies
[ ]	[x]	Eukaryotic cell lines
[x]	[ ]	Palaeontology and archaeology
[ ]	[x]	Animals and other organisms
[x]	[ ]	Human research participants
[x]	[ ]	Clinical data
[x]	[ ]	Dual use research of concern

Methods

n/a	Involved in the study	
[x]	[ ]	ChIP-seq
[ ]	[x]	Flow cytometry
[ ]	[x]	MRI-based neuroimaging

Antibodies

Antibodies used	Primary antibodies:<br>ATP-C (SCMAs) (ab181243; Abcam 1/1000), VDAC (PC548; Calbiochem 1/666), HSP60 (ab46798; Abcam 1/666), PINK1 (sc-33796; Santa Cruz Biotechnology 1/500), NDUFS1 (sc-50132; Santa Cruz Biotechnology 1/500), PFKFB3 (H00005209-M08; Novus Biologicals 1/500), p25/35 (2680; Cell Signalling 1/666), caspase-3 (9661S; Cell Signalling 1/2000), β-Actin (A5441; Sigma 1/30000), cdk5 (sc-6247; Santa Cruz Biotechnology 1/500), lamp1 (1D4B; Developmental Studies Hybridoma Bank 1/100), LC3 (2775; Cell Signaling 1/1000), Parkin (sc_32282; Santa Cruz Biotechnology 1/100), OCT4 (ab19857; Abcam 1/200), SOX2 (AF2018; R&D Systems 1/100), Nanog (ab21624; Abcam 1/100), Tra-1-60 (MAB4770; R&D Systems 1/100), TUJ-1 (MAB1195; R&D Systems 1/200), ATP5A (ab14748; Abcam 1/100), GFAP (G6171; Sigma 1/500), IBA1 (019–19741; Wako 1/500), Nestin (MA1-110; Thermo Fisher 1/100), GFAP (AB5541; Millipone 1/800), B-Tubulin III (ab18207; Abcam 1/500), CD45 (553076; BD 1/200), β-Tubulin III (T2200; Sigma 1/300) and CLN7 (donated by Dr. Stephan Storch 1/500).

Secondary antibodies:
Goat anti-rabbit-HRP (sc-2030; Santa Cruz Biotechnology 1/10000), goat anti-mouse-HRP (170-6516; Bio-Rad 1/10000), rabbit anti-goat-HRP (ab6741-1; Abcam 1/10000), goat anti-rabbit-HRP (170-6515; Bio-Rad 1/3000), Cy2 goat anti-mouse (115-225-003; Jackson Immuno-Research 1/500), Cy3 goat anti-rabbit (111-165-003; Jackson Immuno-Research 1/500), Cy3 donkey anti-rat (712-165-153; Jackson Immuno-Research 1/800), Alexa Fluor 568 goat anti-mouse (A-11031; Invitrogen 1/500), Alexa Fluor 488 goat anti-rabbit (A-11008; Thermo Fisher 1/500), Alexa 488 donkey anti-rabbit (A-31573; Molecular Probes 1/500), Alexa 647 goat anti-rat (A-21247; Thermo Fisher 1/500)

Validation

Antibodies used in this study were validated by the manufacturer who provided references on their websites using the catalog number provided and/or proven to work in the following papers (references belong):
-ATP-C (SCMAs) (ab181243; Abcam 1/1000) was already employed in Centa JL et al. 2020; Wang QJ et al. 2020; among others.
- VDAC (PC548; Calbiochem 1/666) was already employed in Crompton M et al. 1999; Yu, W.H., et al. 1995; among others.
- HSP60 (ab46798; Abcam 1/666) was already employed in Lee Y et al. 2021; Sánchez-Morán I et al. 2020; among others.
- PINK1 (sc-33796; Santa Cruz Biotechnology 1/500) was already employed in Sengupta A et al. 2011; Polletta L et al. 2015; among others.
- NDUFS1 (sc-50132; Santa Cruz Biotechnology 1/500) was already employed in Martin MA et al. 2005; Duncan AM et al. 1992; among others.
- PFKFB3 (H00005209-M08; Novus Biologicals 1/500) was already employed in Almeida A et al 2010; Desideri E et al. 2014; among others.
- p25/35 (2680; Cell Signalling 1/666) was already employed in Shuo Wang et al 2020; Christina Ising et al 2019; among others.
- caspase-3 (9661S; Cell Signalling 1/2000) was already employed in Mi Hye Kim et al 2021; Jasmin Morandell et al 2021; among others.
- β-Actin (A5441; Sigma 1/30000) was already employed in Melanie Si Yan Tan et al. 2019; Lorraine Springuel et al 2014; among others.
- cdk5 (sc-6247; Santa Cruz Biotechnology 1/500) was already employed in Song B et al 2000; Tuo QZ et al 2018; among others.
- lamp1 (1D4B; Developmental Studies Hybridoma Bank 1/100) was already employed in Martinez-Lopez M et al 2016; Garcia-Macia et al 2021; among others.
- LC3 (2775; Cell Signaling 1/1000) was already employed in Martinez-Lopez M et al 2017; Garcia-Macia et al 2019; among others.
- Parkin (sc-32282; Santa Cruz Biotechnology 1/100) was already employed in Nguyen TN et al 2021; Padman BS et al 2019; among others.
- OCT4 (ab19857; Abcam 1/200) was already employed in Trujillo CA et al 2021; Jin X et al 2019; among others.
-SOX2 (AF2018; R&D Systems 1/100) was already employed in Gao X et al 2020; Lazarus KA et al 2018; among others.
-Nanog (ab21624; Abcam 1/100) was already employed in Alici-Garipcan A et al 2020; Chikina AS et al 2019; among others.
-Tra-1-60 (MAB4770; R&D Systems 1/100) was already employed in De Sousa PA et al 2017; Kim SY et al 2012; among others.
-TUJ-1 (MAB1195; R&D Systems 1/200), was already employed in Brodie-Kom J et al 2021; Men Y et al 2019; among others.
-ATP5A (ab14748; Abcam 1/100), was already employed in Jo DS et al 2020; Lieber T et al 2019; among others.
-GFAP (G6171; Sigma 1/500), was already employed in Vinukonda G et al 2012; Tse KH, et al 2014; among others
-IBA1 (019–19741; Wako 1/500), was already employed in Stowell RD et al 2019; Jacob F et al 2020; among others.
-Nestin (MA1-110; Thermo Fisher 1/100), was already employed in Ahn LY et al 2021; Mendivil-Perez M et al 2017; among others.
-CD45 (553076; BD), was already employed in Johnson P et al 1997; van Ewijk W et al 1981; among others.
-GFAP (AB5541; Millipone 1/800), was already employed in Zhang L et al 2020; Lavrov I et al 2016; among others.
-B-Tubulin III (ab18207; Abcam 1/500), was already employed in Choi YS et al 2020; Navneet S et al 2019; among others.
-β-Tubulin III (T2200; Sigma 1/300) was already employed in Nancy S V et al 2018; Hummel T et al; among others.
-CLN7 antibody (donated by Dr. Stephan Storch) was validated in whole cell total membrane extracts of primary cultures of neurons of wild type and CLN7Δex2 mice as shown in Supplementary Figure 1a and previously published in Brandenstein L et al 2016.

Eukaryotic cell lines

Policy information about cell lines

Cell line source(s)	Induced pluripotent stem cells (iPSC) and Neural Progenitor Cells (NPC)
Authentication	Cell line was authenticated by Prof. Sara Mole. The use of human derived cells were approved by the UCL Research Ethics Committee (IRAS Reference number 95005). Written informed consent was obtained from the patients.
Mycoplasma contamination	Cells were tested negative for mycoplasma contamination.
Commonly misidentified lines (See ICLAC register)	No misidentified cell lines were used in this study.

Animals and other organisms

Policy information about studies involving animals; ARRIVE guidelines recommended for reporting animal research

Laboratory animals	Cln7Δex2 mice (PMID: 26681805) in C57BL6/J background were used: Cln7Δex2 or +/+ (WT) genotypes. Animales were crossed with mCATLoxP/+ CAMKIIaCre/+ mice to obtain animals with conditional reduction of mitochondrial ROS (PMDI: 33711713). Male mice were used for in vivo experiments, from 2 to 8 months of age.
Wild animals	No wild animals were used in the study
Field-collected samples	No field collected samples were used in the study.

Ethics oversight	Procedures were approved by the Bioethics Committee of the University of Salamanca and or CIC bioGUNE (PET and MRS) in accordance with the Spanish legislation (RD53/2013).

Note that full information on the approval of the study protocol must also be provided in the manuscript.

Flow Cytometry

Plots

Confirm that:

- [x] The axis labels state the marker and fluorochrome used (e.g. CD4-FITC).
- [x] The axis scales are clearly visible. Include numbers along axes only for bottom left plot of group (a 'group' is an analysis of identical markers).
- [x] All plots are contour plots with outliers or pseudocolor plots.
- [x] A numerical value for number of cells or percentage (with statistics) is provided.

Methodology

Sample preparation	Mice cortical neurons from primary cultures or freshly isolated from brain were used. These cells were treated with the corresponding probes (Mitosox, DilC1),following manufacturer instructions, during a determined time. After incubation with the corresponding probe, the cells were centrifuged and the pellets were resuspended in PBS for further analysis.
Instrument	FACScalibur flow cytometer (BD Biosciences), equipped with a 15 mW argon laser
Software	CellQuest ™ v5 for adquisition and Paint-A-Gate ™ PRO (BD Biosciences) and FlowJo v10 for data quantification.
Cell population abundance	At least 100,000 events were acquired in triplicate and by condition.
Gating strategy	The threshold of the analyzer was adjusted in the corresponding channel of the flow cytometer to exclude most subcellular residues or cellular aggregates in the SSC/FSC plot. The median intensity values were obtained for each sample, and the FMO (unstained cells) substracted.

- [x] Tick this box to confirm that a figure exemplifying the gating strategy is provided in the Supplementary Information.

Magnetic resonance imaging

Experimental design

Design type	single imaging session per animal
Design specifications	minimum of 2 spectra per animal per session. Imaging sessions were repeated after 1 week if spectra did not fit well by the LC Model software.
Behavioral performance measures	N/A

Acquisition

Imaging type(s)	1H-MRS (proton Magnetic resonance spectroscopy) localizaed in voxel
Field strength	11.7 T
Sequence & imaging parameters	PRESS with water suppresion, TE=17 ms, TR = 2500 ms, 2048 points covering a width of 11 ppm
Area of acquisition	ca. 3.5 uL voxel centered in the striatum of the animal, with slight variations in position and voxel individual dimensions to avoid the iclusion of ventricles (CSF) inside the voxel.

Diffusion MRI [ ] Used [x] Not used

Preprocessing

Preprocessing software	LC Model with default parameters
Normalization	N/A
Normalization template	N/A
Noise and artifact removal	N/A
Volume censoring	N/A

Statistical modeling & inference

Model type and settings	N/A
Effect(s) tested	N/A

Specify type of analysis: ☐ Whole brain ☒ ROI-based ☐ Both

Anatomical location(s)	ca. 3.5 uL voxel at the center of the right striatum, avoinding the inclusion of ventricles (CSF)
Statistic type for inference (See Eklund et al. 2016)	N/A
Correction	N/A

Models & analysis

n/a	Involved in the study
☒	☐ Functional and/or effective connectivity
☒	☐ Graph analysis
☒	☐ Multivariate modeling or predictive analysis
